# 2456: Genetic determinants of recovery after mild traumatic brain injury: Can study samples be identified from electronic medical records linked to DNA biobanks?

**DOI:** 10.1017/cts.2017.76

**Published:** 2018-05-10

**Authors:** Jessica Dennis, Scott Zuckerman, Aaron Yengo-Kahn, Nancy Cox, Gary Solomon

**Affiliations:** The Vanderbilt Sports Concussion Center, VUMC, Nashville, TN, USA

## Abstract

OBJECTIVES/SPECIFIC AIMS: To develop an algorithm that identifies post-concussion syndrome (PCS) cases and controls from among patients with mild traumatic brain injury (mTBI) in a large academic biobank. METHODS/STUDY POPULATION: The Vanderbilt University Medical Center’s (VUMC) electronic medical record (EMR) research database includes longitudinal medical record data on 2.5 million people. DNA and genotype data were also available for >225,000 of these individuals. Our algorithm used a combination of billing codes and natural language processing to apply inclusion and exclusion criteria. We defined PCS cases as those with a PCS billing code (ICD-9 310.2 or ICD-10 F07.81) and/or symptoms of PCS within 1–6 months of a qualifying mTBI. We will compare the positive predictive value of our algorithm to that of 2 simpler case selection schemes: (1) 1 instance of the PCS billing code anywhere in the medical record; and (2) 2 or more instances of the PCS billing code anywhere in the medical record. RESULTS/ANTICIPATED RESULTS: An mTBI was diagnosed in 28,720 patients regularly attending VUMC, and 528 of these patients were classified as PCS cases by our algorithm. The characteristics of our EMR sample reflected known risk factors for PCS. Our cases were more likely than controls to be female (49.4% vs. 38.4%), to have sustained a previous TBI (31.0% vs. 12.0%) and to have comorbid mood disorders. Our PCS cases were also more likely than controls to be <18 years of age (42.4% vs. 33.6%) and to have a sports-related keyword associated with the mTBI (44.1% vs. 25.2%), emphasizing the relevance of PCS to young athletes. Nonetheless, the number of PCS cases identified by our algorithm was small, and within the VUMC EMR, there were 5039 patients with 1 PCS billing code, and 2457 patients with 2 or more PCS billing codes anywhere in their EMR. Our next step is to calculate the positive predictive values of each selection scheme by manually reviewing the EMR of a selection of cases. Ultimately, we will implement the selection scheme that maximizes both positive predictive value and sample size, and in future work, we will genotype the selected patients to better understand the genetic architecture of PCS. DISCUSSION/SIGNIFICANCE OF IMPACT: EMR and biobanks are the future of human health research, and we asked whether complex algorithms or simple billing codes were best for studying the genetics of recovery after mTBI within the VUMC EMR. Our results are relevant to other studies of brain injury phenotypes within biobanks, including recovery from moderate or severe TBI, recovery from stroke, or the occurrence of delirium after routine surgery, and will help transform biobanks into fruitful research tools.

